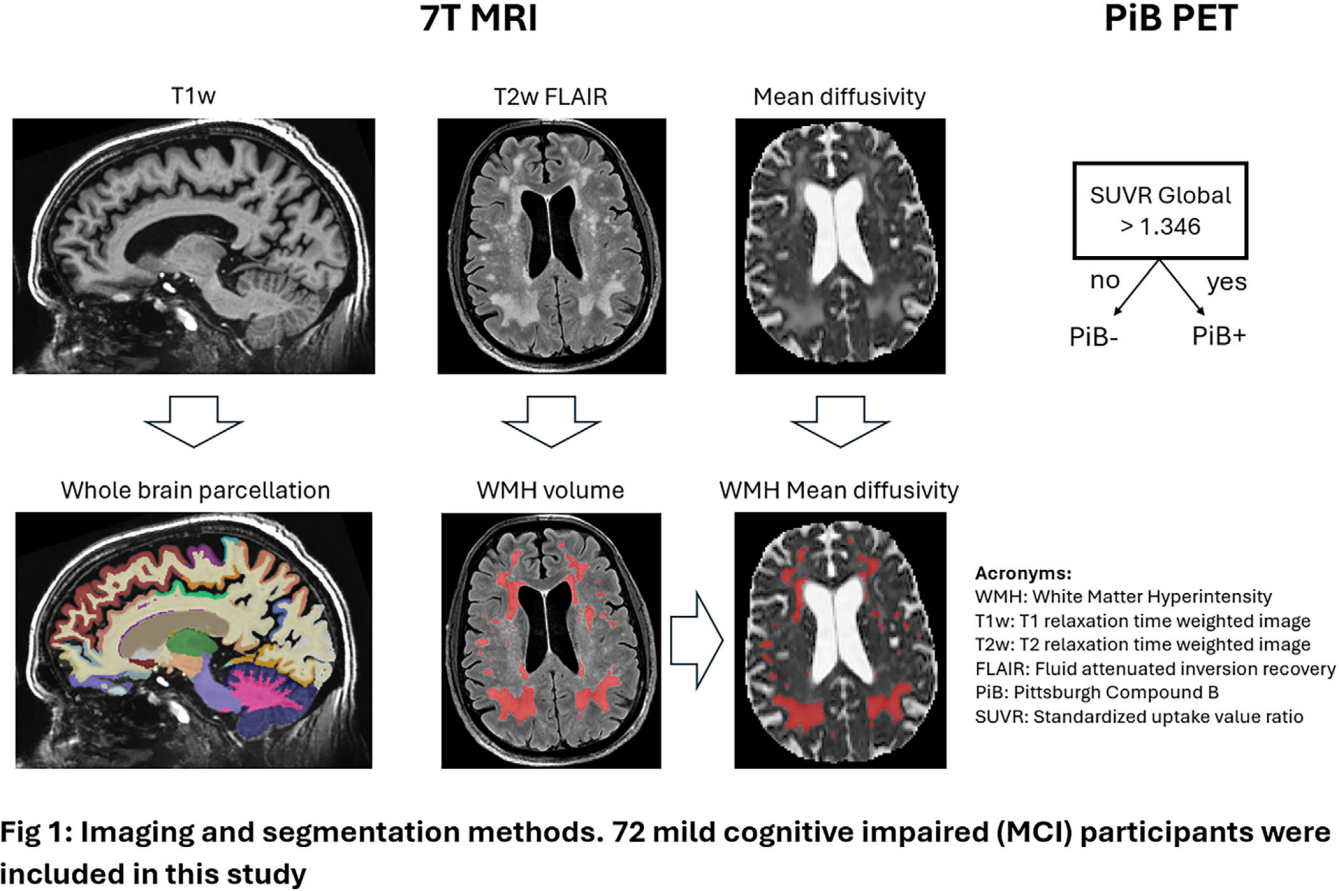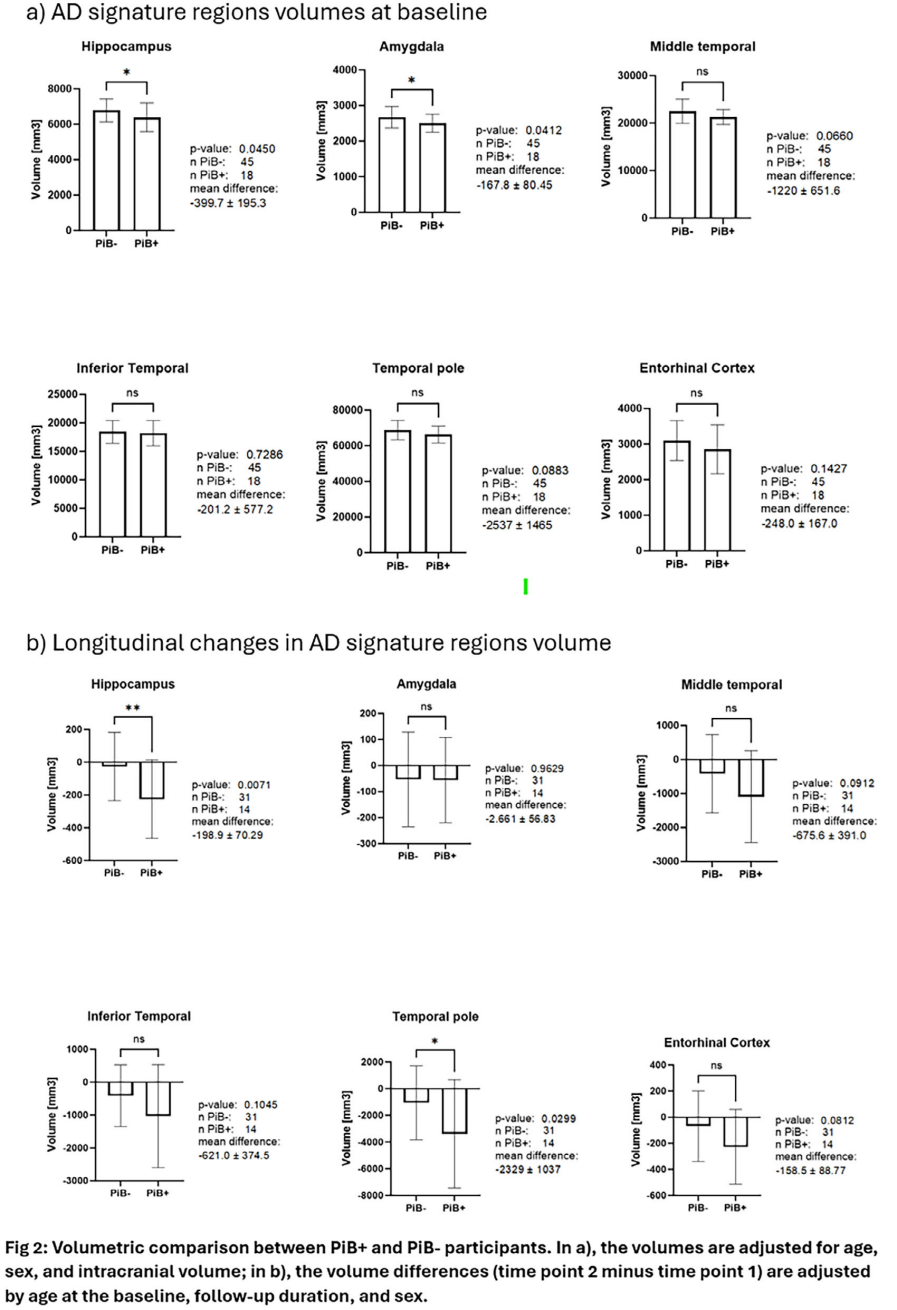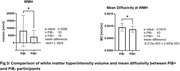# Associations of gray matter volume and white mater hyperintensity mean diffusivity and volume with PiB status in mild cognitive impairment

**DOI:** 10.1002/alz.094140

**Published:** 2025-01-09

**Authors:** Tales Santini, Jinghang Li, James E Emanuel, Jr‐Jiun Liou, Andrea Sajewski, Cong Chu, Bruno de Almeida, Brian J Lopresti, Howard J Aizenstein, Minjie Wu, Ariel Gildengers, Tamer S Ibrahim

**Affiliations:** ^1^ University of Pittsburgh, Pittsburgh, PA USA; ^2^ University of Pittsburgh Medical Center, Pittsburgh, PA USA

## Abstract

**Background:**

White matter hyperintensities are associated with vascular diseases, while amyloid deposition serves as an early indicator of Alzheimer's disease (AD). Despite both pathologies contributing to cognitive decline, the ability to distinguish between them is crucial in selecting the appropriate course of treatment or management of these conditions.

**Method:**

71 mild cognitive impairment (MCI) participants completed the baseline imaging (age 72.6±7.61 years, 38 females) and were included in this study. Pittsburgh Compound B (PiB) PET scans were acquired. 7T MRI images were acquired with a customized radiofrequency coil [1] and included T1w MPRAGE, T2w FLAIR and diffusion sequences. The brain was parcellated using Freesurfer and wmh‐seg [2]. We extracted the baseline (N=63) and longitudinal volumes (N=45, follow up duration = 1.22±0.26 years) of AD signature regions (hippocampus, amygdala, temporal lobes) [3] and the volumes (N=69) and mean diffusivity (N=58) in the white matter hyperintensities and compared the PiB+ (age 75.73±7.97, total SUVR > 1.346, CIRS‐G=8.8±4.1) vs PiB‐ (age 71.5±7.32, CIRS=10.62±3.9) participants. We performed t‐tests adjusting for age, sex, and intracranial volume for baseline variables and adjusting for age at baseline, follow up duration, and sex for the longitudinal variables.

**Result:**

We found a significantly smaller volume in the Hippocampus and amygdala in the PiB+ group and significantly bigger volume of white matter hyperintensities in the PiB‐ group. PiB‐ group also presented higher mean diffusivity inside the white matter hyperintensities, potentially indicating a more advanced stage of the lesion, however partial volume effects need to be evaluated in future work. Longitudinal analysis shows a rapid volume decline in some AD signature gray matter regions in the PiB+ group.

**Conclusion:**

Our results demonstrate the feasibility of high‐resolution and high‐quality 7T MRI in potentially differentiating between neurodegenerative processes related to AD type dementia versus vascular disease in an MCI population.